# Magnetic Resonance-Compatible Arm-Crank Ergometry: A New Platform Linking Whole-Body Calorimetry to Upper-Extremity Biomechanics and Arm Muscle Metabolism

**DOI:** 10.3389/fphys.2021.599514

**Published:** 2021-02-19

**Authors:** Riemer J. K. Vegter, Sebastiaan van den Brink, Leonora J. Mouton, Anita Sibeijn-Kuiper, Lucas H. V. van der Woude, Jeroen A. L. Jeneson

**Affiliations:** ^1^Center for Human Movement Sciences, University Medical Center Groningen, University of Groningen, Groningen, Netherlands; ^2^Department of Biomedical Sciences of Cells and Systems, Cognitive Neuroscience Center, University Medical Center Groningen, Groningen, Netherlands; ^3^Center for Rehabilitation, University Medical Center Groningen, Groningen, Netherlands; ^4^Center for Child Development and Exercise, Wilhelmina’s Children’s Hospital, University Medical Center Utrecht, Utrecht, Netherlands

**Keywords:** 31P-magnetic resonance spectroscopy, arm-crank ergometer, calorimertry, physiology, biomechanics, upper-body exercise

## Abstract

**Introduction:**

Evaluation of the effect of human upper-body training regimens may benefit from knowledge of local energy expenditure in arm muscles. To that end, we developed a novel arm-crank ergometry platform for use in a clinical magnetic resonance (MR) scanner with ^31^P spectroscopy capability to study arm muscle energetics. Complementary datasets on heart-rate, whole-body oxygen consumption, proximal arm-muscle electrical activity and power output, were obtained in a mock-up scanner. The utility of the platform was tested by a preliminary study over 4 weeks of skill practice on the efficiency of execution of a dynamic arm-cranking task in healthy subjects.

**Results:**

The new platform successfully recorded the first ever *in vivo* ^31^P MR spectra from the human biceps brachii (BB) muscle during dynamic exercise in five healthy subjects. Changes in BB energy- and pH balance varied considerably between individuals. Surface electromyography and mechanical force recordings revealed that individuals employed different arm muscle recruitment strategies, using either predominantly elbow flexor muscles (pull strategy; two subjects), elbow extensor muscles (push strategy; one subject) or a combination of both (two subjects). The magnitude of observed changes in BB energy- and pH balance during ACT execution correlated closely with each strategy. Skill practice improved muscle coordination but did not alter individual strategies. Mechanical efficiency on group level seemed to increase as a result of practice, but the outcomes generated by the new platform showed the additional caution necessary for the interpretation that total energy cost was actually reduced at the same workload.

**Conclusion:**

The presented platform integrates dynamic *in vivo* ^31^P MRS recordings from proximal arm muscles with whole-body calorimetry, surface electromyography and biomechanical measurements. This new methodology enables evaluation of cyclic motor performance and outcomes of upper-body training regimens in healthy novices. It may be equally useful for investigations of exercise physiology in lower-limb impaired athletes and wheelchair users as well as frail patients including patients with debilitating muscle disease and the elderly.

## Introduction

Optimal upper-extremity functioning is a prerequisite for healthy performance under different conditions, such as activities of daily living of persons in general and in particular for individuals with lower limb impairments, the work of many industrial laborers, or during different athletic disciplines. Upper-extremity cyclic exercise is more straining and less efficient than leg exercise, while shoulders and wrists are prone to overuse complaints and pain (van der Woude et al., 2001). Whole-body measures of work capacity and physical stress in upper-body exercise monitor the lumped activation of a multitude of diverse and small muscle masses in a considerably more complex functional anatomy compared to lower body exercise (Veeger and van der Helm, 2007). While upper and lower body physical activities roughly follow similar physiological and biomechanical pathways in exercise testing, training, practice and motor skill learning (Sparrow, 2000; Jones and Poole, 2005), absolute and relative outcomes of cardiometabolic stress and efficiency are different.

Whole body physiological outcomes in upper-body exercise (e.g., de Groot et al., 2014; Kraaijenbrink et al., 2017) are limited in their detail in both healthy and clinical populations. To accurately monitor the energetics of upper-body exercise and study adaptations in response to practice or training in healthy and clinical populations, local muscular readouts are additionally needed (Vegter et al., 2014). Such knowledge may advance understanding of physiological and biomechanical mechanisms of function, adaptation and training as well as pathology in this particular mode of human physical activity.

Here, we report on a novel investigative platform for experimental study of upper-extremity cyclic exercise in humans enabling *in vivo* biochemical and physiological data gathering from individual arm muscles as well as ensembles of arm muscles all the way up to the whole body. An existing MR-compatible leg-crank ergometer (Jeneson et al., 2010) was hereto adapted for asynchronous arm-crank ergometry (Dallmeijer et al., 2004; van Drongelen et al., 2009; Powers et al., 2017) and implemented on a multi-nuclear MRI scanner for dynamic *in vivo* ^31^P spectroscopic assay of ATP metabolism in proximal musculature of the human arm. Complementary datasets on whole body oxygen consumption, upper extremity muscle electrical activity, force and power output during arm-crank exercise, respectively, were obtained in parallel experiments in a mock-up MRI scanner. The utility of the new platform was tested in an arm-cycling practice experiment in healthy individuals. Specifically, we tested the hypothesis that moderate intensity practice in naïve test-subjects would result in motor skill learning causing improvements in upper-extremity muscle coordination as well as a reduction in oxidative energy requirement, respectively, during execution of a standardized arm-cranking task (ACT).

## Methods

### Participants

Six able-bodied naive participants were recruited for the study. The physical task of arm-cranking inside the 60 cm diameter bore of a clinical MRI scanner imposed physical constraints on body-height (maximum of 1.80 m) and upper-body width (small to moderately broad). The physical characteristics of the five subjects that completed the study are shown in Table 1; one subject dropped out during the training intervention. Other exclusion criteria were claustrophobia, hypersensitivity to loud noises or presence of metal inside the body. Individuals with prior arm-cycling experience were excluded as the effect of practice is expected to be the highest at the beginning (Kraaijenbrink et al., 2020). It was ensured that the participants had no shoulder impairments or other injuries which could limit them in their ability to perform the cyclic exercise. Subjects were asked to refrain from any major upper-body exercise for the duration of the study. All participants signed informed consent and passed the PAR-Q physical readiness questionnaire before the start of the study (Thomas et al., 1992). The study was approved by the Local Ethics Committee, of the Centre for Human Movement Sciences, University Medical Centre Groningen, University of Groningen, Netherlands.

**TABLE 1:** Characteristics of the study subjects.

	PP 1	PP 2	PP 3	PP 4	PP 5
Age (year)	21	23	26	23	23
Height (m)	1.73	1.67	1.69	1.67	1.76
Weight (kg)	66	63	52	70	69
Arm span (m)	1.76	1.65	1.69	1.67	1.75
Gender	Male	Female	Male	Female	Male

*PP, Participant.*

### MR-Compatible Arm-Crank Ergometry Investigative Platform

**FIGURE 1:**
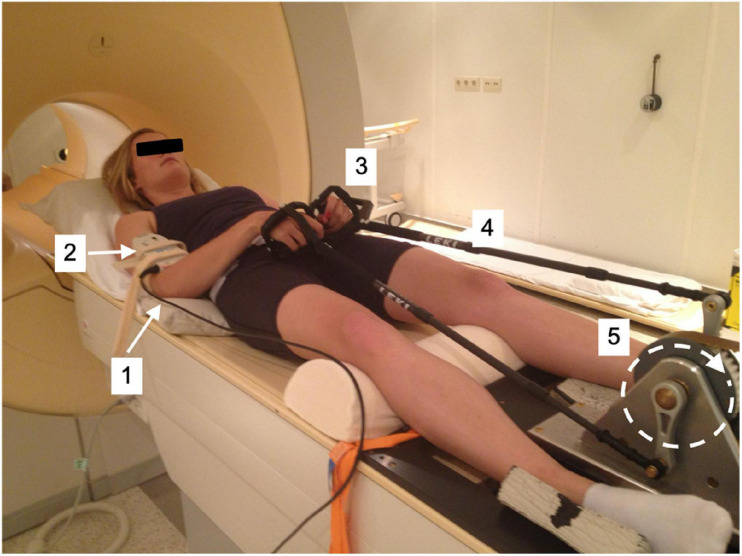
Test subject mounted supine, head-first on patient support of MRI scanner fitted with arm-crank ergometer. [1] stationary elbow joint; [2] ^31^P send-receive surface coil (Philips Healthcare); [3] carbon handgrips; [4] adjustable-length carbon ski poles (Leki, Italy); [5] rotating crank-wheel connected via a nylon belt to a wooden fly-wheel with mechanical brake.

**FIGURE 2:**
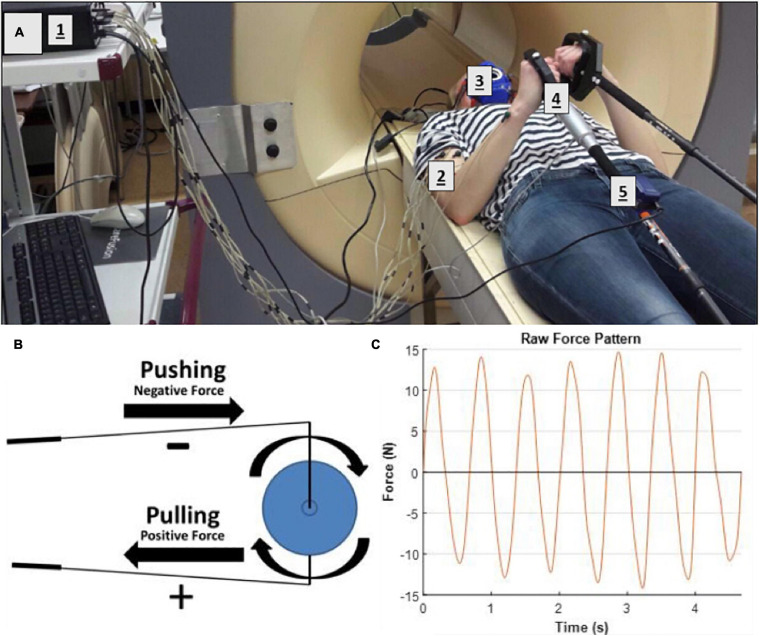
**(A)** Test-subject mounted supine, head-first on patient support of mock-up MRI scanner fitted with arm-crank ergometer. [1] Porti 5 data recording and storage system; [2] EMG electrodes; [3] mask fro Breath-by-breath spirometry; [4] LCM300 load cell of instrumented pole; [5] MT-9 accelerometer attached onto pole. **(B)** Illustration of forces applied to the crank **(C)** excerpt from force recording on the LCM300 load cell during arm-cranking exercise.

A previously described MR-compatible leg-crank cycle ergometer constructed from non-ferrous materials to minimize interference with the static magnetic field of clinical MRI scanners (Jeneson et al., 2010) was refitted for arm-cranking. Two length-adjustable carbon ski poles (Leki, Italy) were hereto fitted with custom-made 3D printed carbon handles and attached to the ergometer cranks using ball joints custom-made from non-ferrous materials. The cranks of the left- and right-hand poles were mounted in an asynchronous mode with the left and right cranks 180 degrees opposed, similar to cycling (Figure 1). An additional pole was constructed and instrumented with a non-MR compatible unidimensional force sensor and accelerometer, respectively, to measure push/pull force output and acceleration of the right arm in a parallel series of experiments conducted in a mock-up MRI scanner replicate (Figure 2; details below). In the latter experiments, subjects were additionally fitted with sets of surface electrodes attached to the *m. Biceps brachii* (*Longus and Brevis*; BB), *m. Brachioradialis* and *m. Triceps* (*Longus and Lateralis*) muscles, respectively, of the right arm as well as a mask for spirometry and a chest strap for heart rate (HR) monitoring (COSMED K4, Italy) enabling dynamic recording of surface EMG (sEMG), breath-by-breath VO_2_ and VCO_2_ and HR data, respectively, in study subjects during ACT execution (Figure 2).

Subjects performed all arm-cranking bouts positioned supine and head-first on the scanner bed (Figures 1, 2). The elbow angle in starting position was 90 degrees in accordance with a maximum torque-angle relationship for the biceps muscle (Pinter et al., 2010). The crank of the right-hand pole was pointing down in this position. This ensured that the very first action to initiate cycling was a pulling motion of the right arm powered by the biceps. At that moment, acquisition was switched from “automatic” to “cycling triggered (van Brussel et al., 2015). Subjects were instructed to perform arm-cycling exercise at 90 rounds per minute (rpm) guided by a metronome. A brake weight of 0.2 kg was applied to the flywheel yielding a workload of 15 W at this crank rate (Supplementary Figure S1). This particular workload was thought to corresponded to “moderate intensity” in healthy subject, since a pilot with one healthy subject showed maximal sustained power-output at 90 rpm was 25–30 W (unpublished data).

### Experimental Design of the Practice Intervention

**FIGURE 3:**
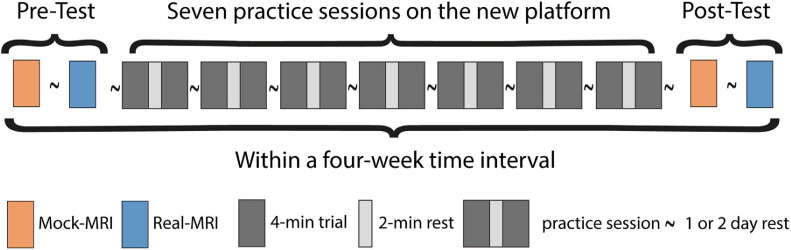
Experimental design of the low-intensity arm-crank practice intervention. Subjects performed a standard arm-cranking task (ACT; see section “Methods”) prior to and following 3 weeks of practice, consisting of 7 practice sessions with a 1 or 2 day res in between. For the pre- and post-training tests, subjects performed two bouts of ACT: one inside a mock-up MRI scanner (orange blocks) and a second bout inside an MRI scanner (blue blocks), respectively, to facilitate acquisition of a comprehensive dataset including bodily oxygen consumption, upper arm muscle activity, force, *in vivo* ATP turnover and pH changes in biceps muscle.

Subjects were distributed over two groups that were studied sequentially over a time span of 8 weeks [4 weeks per group, protocol based on (De Groot et al., 2002; Vegter et al., 2014)]. Each group of three subjects visited our center in their first week of the study on 2 consecutive days for the pre-test trials (Figure 3), followed by a total of seven practice-sessions during weekdays, with a minimum of 1 day rest in between. Each practice session consisted of 2 × 4 min bouts of supine arm-cranking at 15 W power output with 2 min of rest between bouts. Following practice, a post-test was recorded in similar fashion to the baseline recordings. Pre- and post-test trials #2 were conducted inside a multi-nuclear 3T Achieva Intera MRI scanner (60 cm bore diameter; Philips Healthcare, Best, Netherlands; Figure 1). Pre- and post-test trials#1 as well as all training sessions were performed inside the mock-up replica of the MRI scanner (Figure 2).

### *In vivo* ^31^P-MRS Data Collection and Processing

*In vivo* ^31^P-MRS measurements were performed during pre-test #2 and post-test #2. A 6 cm diameter, single-turn send-receive ^31^P surface coil (P60; Philips Healthcare, Best, Netherlands) was positioned over the BB muscle of the right arm of the study subjects. The coil was manually tuned and matched at the Larmor frequency of phosphorus-31 at 3 Tesla (51.73 MHz). Free induction decays (FIDs) were next acquired using a 90-degree adiabatic excitation pulse and characterized by sampling 2,048 points with a dwell time of 0.33 ms. Firstly, a measurement of the basal phosphorus metabolite levels in the biceps muscle was performed (16 averaged FIDs; repetition time (TR) 13,333 ms). During exercise, continuous MR spectroscopic data gathering from the BB muscle was synchronized with the arm-cranking frequency as described elsewhere (van Brussel et al., 2015). Here, FIDs were recorded with a TR of 2,666 ms (corresponding to 4 rotations of the handcycle at 90 rpm). Four FIDs were averaged per individual spectrum and stored for off-line analysis.

FIDs were analyzed in the time domain with respect to resonance amplitudes and frequencies of ATP, inorganic phosphate (Pi), phosphocreatine (PCr), and phosphomonoesters (PME) and quantified using the AMARES (Advanced Method for Accurate, Robust, and Efficient Spectral fitting) algorithm in the jMRUI software package (version 3.0) using custom prior knowledge of starting values, line width, frequency and shape of the resonance peaks (Vanhamme et al., 1997). Intramuscular pH was calculated from the difference in resonance frequency between Pi and PCr [in parts per million (ppm)] as described elsewhere (Tobin et al., 1972; Prompers et al., 2006). For each subject, residual biceps PCr content (% of resting) and intramuscular pH in the final minute of ACT execution were determined as physiological outcomes.

### sEMG Data Collection and Processing

Surface EMG (sEMG) recordings were collected from the participants’ right arm during pre-test 1 and post-test 1 using the Porti-5 system. The Porti-5 system measured EMG activity in mV at a sample frequency of 1,600 Hz, PortiLab2 software was used for data acquisition. The skin of the participants was shaved, scrubbed and cleaned with alcohol to improve EMG electrode conductivity. After evaporation, two EMG electrodes were patched upon the muscle belly of interest with approximately 0.02 m distance between the center of the electrodes (Cleartrode, ConMed, United States). The following six muscles were, respectively, patched with electrodes and connected to the Porti system; *m. biceps brachii longus and brevis, triceps longus, triceps lateralis*, and *brachioradialis*. Subjects were next asked to take place in the starting position of the handcycle trial and perform a total of six Maximum Voluntary Contractions (MVCs) on the handgrip equipped with the LCM300 load cell while the flywheel of the ergometer was locked. Each MVC lasted 5 s and the participants had 2 min of rest between each MVC. Three MVCs of elbow flexion were performed followed by three MVCs of elbow extension.

Raw sEMG recordings were processed using custom written scripts in Matlab. sEMG signals were filtered using a fourth order dual pass Butterworth filter setting. A high pass filter with a cut-off frequency of 6 Hz was performed on the raw sEMG signal to remove low frequency noise. Subsequently, full wave rectification was performed by taking the absolute value of the signals. The rectified signal was low-pass filtered using a cut-off frequency of 10 Hz to create the linear envelope of the sEMG signal. sEMG signal amplitudes during ACT execution were determined for each muscle and normalized to maximal amplitude recorded during MVC of elbow flexion (biceps and brachioradialis) or extension (triceps) yielding values in units of “percentage of maximal” (%). Maximal amplitude was calculated as the mean of three 500 ms sEMG recordings during MVC around the time point at which force on the load cell reached its peak (0.125 s before the peak till 0.375 s after the peak).

### Force-Sensor Data Collection and Processing

An additional pole was constructed and instrumented with a non-MR compatible unidimensional force sensor (LCM300 load cell; Futek, Irvine, CA, United States), and a MT-9 accelerometer (Xsens, Enschede, Netherlands). Each device was connected to a Porti 5 system (TMS International, Enschede, Netherlands) to record its millivolt (mV) outputs with a sample frequency of 1,600 Hz, yielding data characterizing of both pushing and pulling forces and pole acceleration in X, Y, and Z direction, respectively. Prior to recordings during ACT execution, baseline mV output of the load cell during 10 s in the static was recorded and nulled. The post-practice datasets of two subjects (#2 and #4) recorded during ACT execution were corrupted due to faulty wiring to the Porti-5 system.

Raw signals from the LCM300 load cell were processed using custom-written Matlab code. First, the signal was filtered using a fourth-order low-pass Butterworth filter at a cut-off frequency of 6 Hz to remove any movement noise and subsequently transformed to N using prior calibration with weights. A typical example of the measured force output is shown in Figure 2. The mean and standard deviation of the height and width of the positive and negative peaks in the force signal of the whole trial were calculated. These values were used to illustrate the average unilateral force signal through the pole onto the crank of arm-cycle. Cubic spline data interpolation was used to create a smoothened pattern between the calculated average peak heights and widths.

### Indirect Calorimetry

The mobile Spirometer Cosmed K4B2 (COSMED, Italy) was used to record gas exchange dynamics during ACT execution. Heart rate data was collected using a strap and software provided by the same supplier. Data were collected during a 10 min rest period followed by the 4 min ACT and a 10 min long recovery period.

VO_2_ (L/min) and VCO_2_ (L/min) recordings were used to calculate the internal energy expenditure (Garby and Astrup, 1987a). Gross mechanical efficiency (ME) was determined using the calculated time-weighted average of internal energy expenditure over the final minute and the known workload of the arm-cycle. Calculations of the weighted average of heart rate and ME, were performed using custom written Matlab scripts (Matlab R2017a, The Mathworks, Natick, MA, United States) (Vegter et al., 2013b).

### Statistics

Only descriptive statistics are reported, since the small number of participants and high number of possible statistical comparisons would result in very low statistical power. On the other hand, the individual outcomes and differences thereof are used to show the potential of the methodology for future research.

## Results

### *In vivo* ^31^P MRS Recordings of m. Biceps Energy- and pH Balance During ACT Execution

**FIGURE 4:**
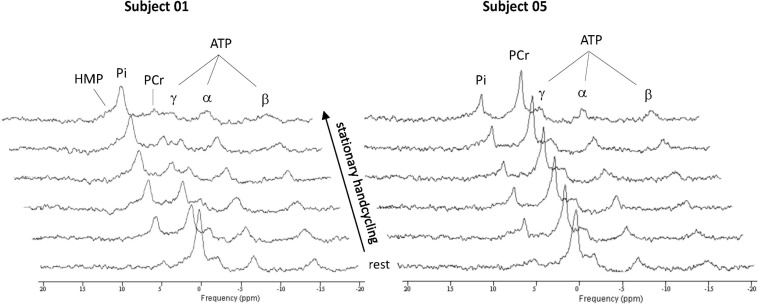
Stack plots of *in vivo* ^31^P MR spectra recorded from them. biceps brachii of two subjects showing widely different changes in intramuscular energy and Ph balance, to generate overall power-output of 15 W required to propel the flywheel at 90 rpm. Each spectrum represents a 11 s time-average recording from the biceps muscle. ATP, adenosine triphosphate; Pi, inorganic phosphate; PCr, phosphocreatine; HMP, hexose monophosphate.

Figure 4 shows time series of *in vivo* ^31^P MRS spectra of the BB muscle of the right arm recorded during ACT execution in two different individuals. Both series showcase the expected inverse changes in PCr and Pi content of the BB muscle in response to the stepwise increase in ATP turnover after arm-cranking was initiated. However, the magnitude of change in intramuscular levels of these ATP metabolites as well as intramuscular pH varied starkly between these individuals. Specifically, in one subject PCr was almost completely depleted at the end of exercise while the median resonance frequency and linewidth of the Pi signal indicated that the BB muscle was severely acidified at that point (pH 6.3 vs. 7.1 in resting biceps) (Figure 4, left stack). The ancillary detection of significant signal upfield from the broad Pi resonance toward the end of exercise in this subject evidenced millimolar intramuscular accumulation of hexose monophosphates (HMP) (Figure 4, left stack). In contrast, PCr depletion, Pi accumulation and acidification of BB muscle fibers recruited during ACT execution in the other subject were all minor (Figure 4, right stack). Together, these datasets represented two extremes of the metabolic response to ACT execution in the BB muscle that we observed within the group of subjects enrolled in the present study. An overview of the individual metabolic responses in terms of magnitude of PCr depletion and intramuscular acidification of the BB muscle during ACT execution and the effect of the practice intervention is presented in Table 2.

**TABLE 2:** Overview of the effects of the practice intervention on physiological outcomes of ACT execution at proximal arm muscle vs. whole body level.

	Proximal arm muscle outcomes						Whole body outcomes							
Subject	Residual BB PCr at end of ACT (% of rest)		Change in BB pH during ACT		sEMG amplitude during ACT (% of MVC)		VO2 (L/Min)		VCO2 (L/Min)		Change in HR during ACT (bpm)		Mechanical efficiency of ACT (%)	
	Pre	Post	Pre	Post	Pre	Post	Pre	Post	Pre	Post	Pre	Post	Pre	Post
#1	5	10	−0.8	−0.5	13	10	0.56	0.56	0.65	0.53	18	13	5.6	8.6
#2	8	7	−0.2	−0.2	11	13	0.63	0.49	0.55	0.45	25	17	5.0	8.3
#3	11	62	−0.5	−0.1	12	7	0.44	0.51	0.51	0.51	26	**22**	6.1	6
#4	12	7	−0.1	−0.4	26	11	0.66	0.46	0.63	0.39	60	24	4.3	10.7
#5	61	58	−0.1	−0.3	5	4	0.54	0.49	0.46	0.38	17	12	7.6	9.3
Mean ± *SD*	19 ± 23	29 ± 29	−0.3 ± 0.3	−0.3 ± 0.2	13 ± 8	9 ± 4	0.57 ± 0.09	0.51 ± 0.04	0.60 ± 0.08	0.45 ± 0.07	29 ± 18	17 ± 5	5.7 ± 1.2	8.6 ± 1.7

*BB, biceps brachii; PCr, phosphocreatine; ACT, arm-cranking task; sEMG, surface electromyography; HR, heart rate.*

### sEMG Activity of Proximal Arm Muscles During ACT Execution

**FIGURE 5:**
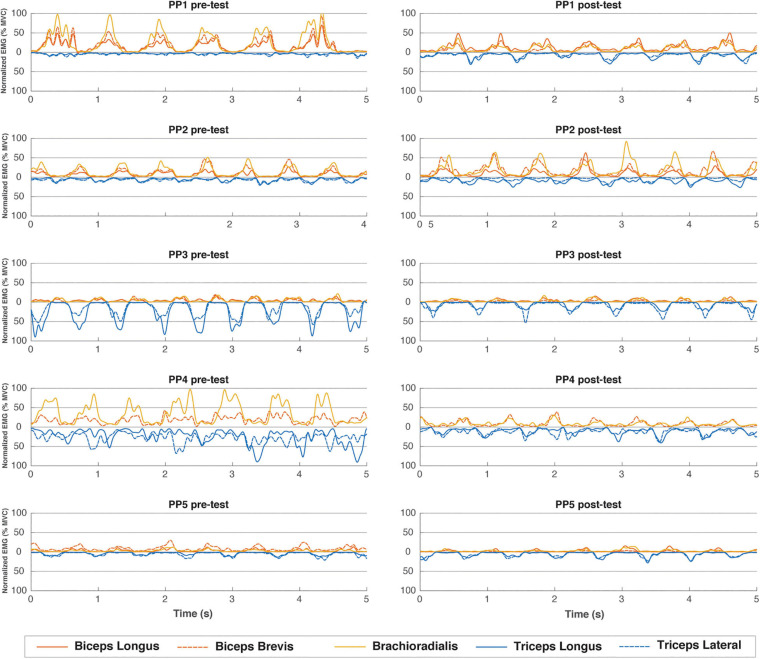
EMG muscle activation pattern (%MVC) over 5 representative seconds during the end of the first minute of the pre- and post-training tests for all individual subjects. Flexor muscles have positive values, extensor muscles are mirrored and depicted as negative values.

Our pre-training sEMG recordings during ACT execution revealed that individual subjects employed distinctly different upper arm muscle recruitment strategies to generate sufficient power output to perform the task (Figure 5, left column). Specifically, participants were found to either predominantly use elbow flexor muscles (participant 1 and 2), elbow extensor muscles (participant 3) or a combination of both (participant 4 and 5), to perform the physical task. When comparing pre- vs. post-practice sEMG recordings, we found that motor skill training did not result in any major change in individual muscle recruitment strategy during ACT execution except for subject #1 (Figure 5, right column). Post-practice amplitudes of sEMG signals recorded during ACT execution from dominantly recruited proximal arm muscles in subjects #1, #3 and #4 were reduced compared to pre-practice (flexor, extensor and mix, respectively, Figure 5). No such change in sEMG amplitude during ACT execution after the practice intervention was observed in subjects #2 and #5 (Figure 5).

### Mechanical Force Output of the Arm During ACT Execution

**FIGURE 6:**
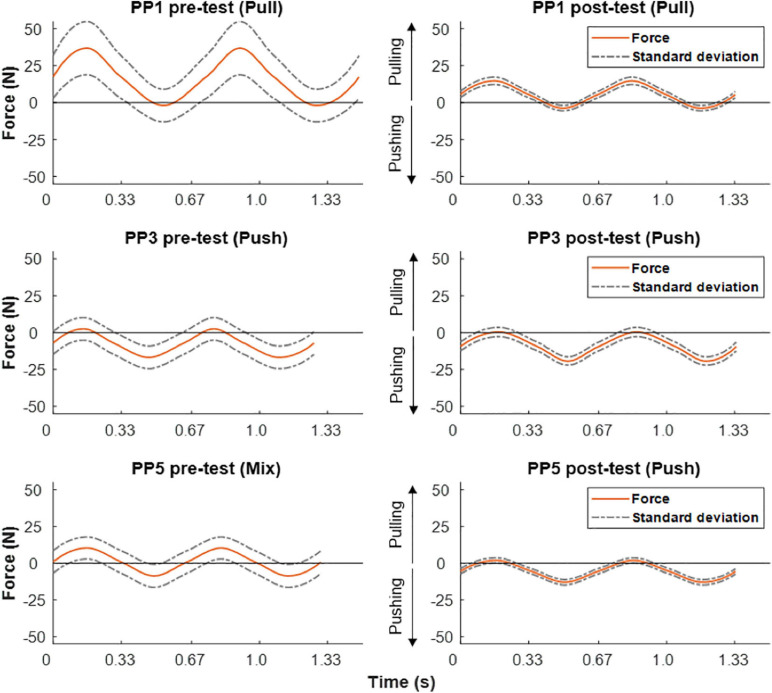
Force output during ACT recorded in pre- (left) vs. post-training test (right) in three test-subjects. Positive forces equal pulling and negative forces equal pushing. From top to bottom different individual strategies in terms of pulling, pushing or mixed, respectively, are clearly discernible.

Figure 6 shows pre- vs. post-practice comparisons of the average dynamics of mechanical force output of the right arm during ACT execution for three individuals. In each case, two complete cycles were averaged over the whole trial. In line with the EMG recordings shown above (Figure 5), subject #1 mostly produced net positive forces during pre-training execution of the handcycling task indicating pulling (flexor) movements on the load cell, while subject #3 mostly produced net negative forces indicate pushing (extensor) movements (Figure 6, left column). Subject #5 evenly alternated pulling and pushing during handcycling in the pre-test (Figure 5, left column). The training intervention resulted in all three subjects in improved reproducibility of mechanical force generation during handcycling as evidenced by a twofold or more reduction of variance (Figure 6, right column). The force recordings in subject #5 suggest that the training intervention resulted in a slight shift from a mixed push-pull strategy toward a predominantly push strategy (Figure 6).

### Comparison of Whole Body vs. Proximal Arm Muscle Outcomes of ACT Execution

**FIGURE 7:**
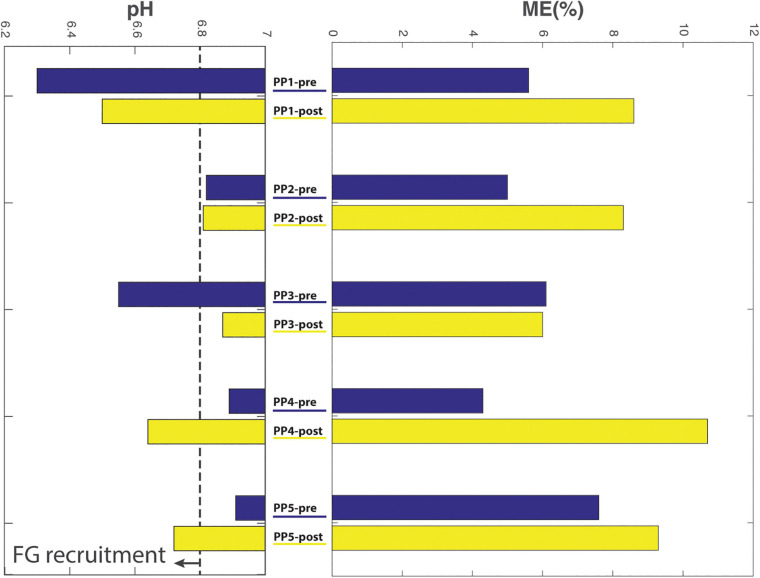
Comparison of biceps acidification during ACT execution (left) vs. gross mechanical efficiency (right; %) of ACT execution during the last minute of exercise, determined pre- vs. post-practice (blue and yellow bars, respectively) for individual subjects.

A comparison of pre- and post-practice findings with respect to whole body outcomes (delta heart rate (ΔHR) and mechanical efficiency (ME)) vs. proximal arm muscle outcomes (end-exercise BB energy- and pH balance and total proximal arm muscle EMG activity) of ACT execution in individual subjects is presented in Table 2. With respect to heart-rate, and total EMG area a reduction because of practice seems present while producing the same workload, which also seems reflected in the increased mechanical efficiency. On the other hand, comparison of these results did not correlate well with the end-exercise BB energy- and pH balance measured with ^31^P-MRS. This is better exemplified in Figure 7, which shows the large individual differences in aerobic and anaerobic contribution to perform the given workload.

## Discussion

The primary innovation of the experimental platform for investigation of upper-extremity cycling exercise in humans is that it uniquely enables *in vivo* data gathering on energy metabolism and pH balance in proximal muscles of the arm performing a calibrated concentric exercise task. Two previous applications of *in vivo* ^31^P MRS to the investigation of energy metabolism in proximal muscle of the human arm employed either a qualitative isometric task (Shields et al., 2015) or no exercise (Rodenburg et al., 1994). Among other applications, the platform can be used to inform upper-body training efficacy evaluations on contributions of aerobic vs. anaerobic ATP production to energy balance in individual proximal arm muscles in relation to whole body energy utilization during exercise. Below, we will discuss how this new information impacted the outcome of a first showcase study of such a practice intervention in healthy naïve subjects. First, technological and practical aspects of the platform including recommendations for future upgrading and application are discussed.

### Technological and Practical Aspects of the Platform

#### *In vivo* ^31^P MRS Recordings From Upper Arm Musculature

The principal practical problem we had to overcome to robustly collect ^31^P MRS data from the human biceps brachii muscle during high-frequency arm-cycling with ∼10 s time resolution and adequate signal-to-noise and resonance linewidth quality for quantitative analysis was the anatomy of human subjects in relation to MRI scanners. Specifically, the biceps muscle is physically on the periphery of the human body, typically some 25 cm out of the central body axis in lean adults, whereas the default “sweet spot” of static field magnetic homogeneity of 3T MRI scanners is typically a 20–30 cm diameter sphere in the center of the magnet. In addition, the inner-diameter of the bore of commonly available clinical MRI scanners including the MRI scanner used in the present study is typically 60 cm. Together, these features constrained both the attainable quality of local static magnetic field homogeneity (“shim”) over the biceps muscle (typical PCr linewidth 30–40 Hz compared to 5–10 Hz in *in vivo* ^31^P MR spectra of medial quadriceps of the upper leg (Schmitz et al., 2010) as well as the physical space for dynamic arm exercise inside the magnet. Any additional signal quality deterioration introduced by motion artifacts during high-frequency arm-cycling (i.e., 90 rpm) was surprisingly minor. We attributed this to the fact that the upper arm remains in one and the same position during arm-cycling around the elbow (Figure 1). In addition, ^31^P MRS data acquisition from the biceps during exercise was synchronized with cycling phase using a custom-built triggering setup described elsewhere (Jeneson et al., 2010). Together, this enabled robust dynamic ^31^P MRS data gathering from the biceps muscle during high-frequency arm cycling with 11s time resolution in all subjects (Figure 4).

### sEMG and Force Sensor Recordings

Upper-arm muscle recruitment patterns in participants performing the arm-cranking task were well reflected by the results of both sEMG and force-sensor recordings during ACT execution (Figures 5, 6, respectively). Of these, sEMG is commonly available in human movement research labs evaluating cyclic upper-body exercise (Abbasi Bafghi et al., 2008; Litzenberger et al., 2015; Quittmann et al., 2018). The force-sensor/pole ensemble was custom-built using an “off the shelf” one-dimensional sensor incorporated in the pole, whereby the pole handle was constructed such that no torque was possible (Methods; Figure 2). Although the force-sensor recordings lacked the detail on individual muscle contributions to overall arm power-output provided by sEMG (Figure 5), the former adequately distinguished flexor from extensor movements with superior signal-to-noise and superior dimensionality (i.e., *N*; Figure 6). Moreover, while similar pictures on arm muscle recruitment during ACT execution in individual subjects emerged from the sEMG and force-sensor recordings in the present study, the latter suggested training induced a small shift from “mixed push/pull” to “pull” mode in subject #5 that was less evident from the sEMG recordings.

### Recommendations for Future Platform Upgrading and Use

The present study employed a conventional single element ^31^P surface coil available from the vendor (Philips Healthcare) to record *in vivo* ^31^P MR spectra from the biceps muscle. In light of our findings of inter-individual differences in pull- vs. push- handcycling strategies, future studies should also collect MRS data from the triceps muscle and preferably simultaneously from both muscles. In principle, use of a two-element ^31^P surface coil in combination with dual receive channels should render simultaneous MRS data collection from both upper-arm muscles feasible. Modern clinical 3T MRI scanners with multinuclear capability from major vendors typically support use of such advanced coil designs. Moreover, these scanners are typically also available in a 70 cm diameter bore size. This both greatly enhances room for in-magnet exercise as well as relaxes some of the constraint on physical dimensions of study subjects that we encountered.

All sEMG, spirometry and mechanical force data recordings were conducted in a parallel series of pre- and post-training tests in a mock-MRI scanner due to issues of MR-compatibility of equipment. Ideally, all these recordings should be done concomitantly with the MR measurements. MR-compatible approaches to collect VO_2_ and VCO_2_ data are available including Douglas bag-based methodologies (McLaughlin et al., 2001; Rietjens et al., 2001; Voorhees et al., 2005) or mass-spectrometry (Whipp et al., 1999). Simultaneous acquisition of sEMG and ^31^P MRS data from the upper arm muscles are also well feasible provided non-gradient-based data acquisition sequences such as used in the present study are employed (Vestergaard-Poulsen et al., 1994). Similarly, the applied torque over the cycle of the crank of the MRI-compatible handcycle ergometer should ideally also be measured in the MRI scanner, and in both arms. While such systems are available and have been used for arm-cranking and hand-cycling studies (Quittmann et al., 2018; Vegter et al., 2019; Kraaijenbrink et al., 2020), these are as of yet MRI-incompatible. The sole available industry-standard MRI cycle ergometer (Lode BV, Groningen, Netherlands) could potentially be fitted with hardware and software to render such measurements feasible in MRI scanners in the future (Lode BV, personal communication).

Lastly, the new platform for upper-extremity cycling exercise presented here may be useful for clinical investigations of a range of study populations and objectives. Firstly, it may contribute to guide training of athletes relying on upper-body activities including rowing, arm-cycling, sailing, rock-climbing or wheelchair track athletes. In these sports the upper-body is highly trained, yet little remains known about fiber-type distributions and relative contributions of aerobic and anaerobic energy production optimal for each particular athletic activity, for upper- and lower-extremities alike (Van Der Zwaard et al., 2018). Secondly, the methodology may also be useful in the field of rehabilitation of individuals with spinal cord injury. These often-traumatic injuries render individuals heavily dependent on their upper-body for daily activity, while there may be a large heterogeneity in upper-body fitness and skill prior to the accident. Of specific interest are any adaptation processes taking place at muscular levels as well as to local and bodily physiology as a consequence of continuous practice and use of their upper-body (Van Der Scheer et al., 2017). Other potential clinical application of the methodology may be in care for patients with neuromuscular disease, primary metabolic myopathies including mitochondrial myopathies as well as secondary myopathies including heart failure and COPD. Here, the key asset is the fact that arm-cranking constitutes a relatively moderate intensity dynamic exercise paradigm (dynamic range 3–30 W at 90 rpm; Supplementary Figure S1) compared to conventional testing using bicycle ergometers. As such, it offers a platform to evaluate the quality of their muscles with respect to mechanical and metabolic functions and monitor the effects of training, pharmaceutical and dietary therapy.

### Outcome of the Practice Intervention

The practice intervention study that we conducted to showcase the new platform tested the hypothesis that 3 weeks of practice would improve the gross mechanical efficiency of execution of a physical task consisting of supine asynchronous arm-cranking at 90 rpm for 6 min against a workload of 15W. Tested solely against the combined results of the conventional physiological measures that we collected, this hypothesis was not rejected: the mean gross ME of ACT execution improved in 4 out of 5 participants after training in the study test-population. The ME of the arm cranking task was lower than previous arm ergometer studies (Kang et al., 1997; Simmelink et al., 2015). In comparison with regular arm cranking, the designed Magnetic Resonance-compatible arm-crank ergometer puts more constraints on the possible movement. Because the elbow joint is in a more-or-less fixed position, elbow flexion-extension is the predominant causes of crank-movement. Hence the shoulder and trunk do not perform work, whereas in regular arm-crank exercise this is the case. We hypothesize that the lower muscle mass active is a possible explanation for the lower mechanical efficiency (van der Woude et al., 2001).

The *in vivo* ^31^P MRS recordings from biceps muscle during ACT execution that we additionally collected, however, showed that, with respect to this particular physiological outcome, the study was biased by results in subjects #4 and #5. Specifically, increased BB acidification during post-practice ACT execution in these individuals (Figure 7) indicated that they had used more fast-twitch fibers with low oxidative capacity (FG fibers; Meyerspeer et al., 2020) to perform the task than at baseline. Neither of these subjects had appreciably changed their arm-cranking strategy toward any “pull” mechanism in response to practice; if any, subject #5 had rather adopted more of a “push” strategy (Figure 6). As a result, ME—i.e., the ratio of whole arm power-output and whole-body aerobic energy expenditure during ACT execution was skewed toward a higher ratio post-practice (Table 2).

This particular finding was similar to results of a study of the effect of a low-intensity training intervention in manual wheelchair propulsion in naïve subjects (Vegter et al., 2015). The authors reported improvement of gross ME of wheelchair propulsion concomitant with opposite rather than parallel changes in power-output over time for the *biceps* and *brachialis* muscles of the upper arm. Notably, power-output of the *biceps* muscle was found to increase, not decrease with training (Vegter et al., 2015). Together, these studies indicate that findings of increased ME after training of any form of upper-body exercise based solely on whole body measurements of oxygen consumption should be interpreted with caution. The new experimental platform for upper-extremity cyclic exercise presented in this report uniquely affords to gather complementary data on energy expenditure in upper-extremity muscles to strengthen objective evaluation of the outcome of upper-body training interventions.

The results of the practice intervention study also provide new insight into the contribution of aerobic vs. anaerobic ATP metabolism in muscles recruited during cyclic upper-body exercise. As discussed in the above, the data showed that the assumption of strictly aerobic muscular ATP metabolism at moderate-intensity dynamic upper-body exercise implicit in the use of the parameter “ME” to evaluate training outcome (Garby and Astrup, 1987b; Almasbakk et al., 2001) did not hold for the majority of untrained, able-bodied, healthy individuals that participated in the present study. Secondly, we found that the contribution of anaerobic motor units of the biceps to power-output during arm-cycling was not uniformly affected by the training intervention across test subjects. Inter-individual differences in learning efficacy have previously been described (Bouchard and Rankinen, 2001; Withagen and van Wermeskerken, 2009; King et al., 2012; Kostrubiec et al., 2012; Vegter et al., 2013a; Vegter et al., 2014) and may become more explicitly characterized using this platform.

### Limitations and Outlook

The novelty of the current approach comes with some technological limitations that should be considered when assessing the results. First, subjects performed the ACT trial twice—i.e., inside the mock-up MRI scanner and inside the MRI scanner, respectively,—both pre- as well as post-training in order to obtain a complete set of experimental data consisting of sEMG, mechanical force and PO, spirometry and ^31^P MRS recordings, respectively (Figure 3). As such, it was assumed that individual participants performed the physical task in both environments in identical manner. Past findings that motor skill acquisition takes place on a timescale of multiple minutes (Vegter et al., 2013a) suggests that this assumption may perhaps have been problematic. However, our results showed that individual arm-cycling strategies were consistent over the entire course of the experiment and did not majorly change during training. On a similar note, only the right side of a bimanual task was measured, hence the distribution of work between both hands could have changed over time and/or differ between participants. Again, the novelty of the method did not allow us to measure both sides yet, but handcycling literature suggest that overall a symmetrical workload is to be expected (Kraaijenbrink et al., 2020).

Secondly, our practice intervention study employing the new platform was limited in its sample-size resulting in low statistical power of evaluation of the study outcome for the group. Here, both the high cost as well as limited availability of MRI scanner time played a role. However, the observed heterogeneity in individual ACT strategy within these five subjects in and by itself suggests that a common group pattern of task execution may perhaps not be expected. On the other hand, past evidence suggests that motor skill acquisition is a general principle of motor functioning and, as such, should hold for groups of subjects (Sparrow et al., 2000; Almasbakk et al., 2001; Lay et al., 2002). Besides including more participants, future studies might clearly define the basal state of each participants and as a consequence individualize their workload. Factors, such as aerobic capacity and muscle mass may have contributed to a relatively different workloads between participants. For example, using a ramp or incremental test to choose a workload based on the responses may be appropriate, as often implemented in VO_2_ studies (Kouwijzer et al., 2019). Also, depending on the research question and ethical approval physical activity in between practice could be monitored.

## Conclusion

The newly developed platform has produced the first ever *in vivo* data on human biceps energy- and pH balance during dynamic exercise in the context of biomechanical and system physiological outcomes. Integration of these measurements with whole body calorimetry, surface electromyography and mechanical measurements during arm-cycling training intervention showed that the study outcome of improved mechanical efficiency post-training was biased by variable contributions of anaerobic motor units of the biceps to power-output during arm-cycling among these novice subjects. This methodology may aid design and evaluation of upper-body training regimens for athletes, wheelchair-bound individuals and patients with debilitating muscle disease.

## Data Availability Statement

The raw data supporting the conclusions of this article will be made available by the authors, without undue reservation.

## Ethics Statement

The studies involving human participants were reviewed and approved by the Local Ethics Committee, of the Centre for Human Movement Sciences, University Medical Centre Groningen, University of Groningen, the Netherlands. The patients/participants provided their written informed consent to participate in this study. Written informed consent was obtained from the individual(s) for the publication of any potentially identifiable images or data included in this article.

## Author Contributions

RV, SB, LW, and JJ contributed to the conception and design of the work, the acquisition, analysis, and interpretation of data and drafted the work. LM contributed to the conception and design of the work, interpretation of data and drafted the work. AS contributed to the design of the work, the acquisition, analysis, and interpretation of data. All authors contributed to the article and approved the submitted version.

## Conflict of Interest

The authors declare that the research was conducted in the absence of any commercial or financial relationships that could be construed as a potential conflict of interest. The reviewer MM declared a past co-authorship with one of the authors JJ to the handling editor.

## Supplementary Material

Supplementary Figure 1 The rolling resistance of the platform is manipulated by adding mass to a friction dependent brake on the flywheel. The consequent RPM-dependent power output (W) was measured by the in-factory lode calibrator and shows good linearity within a range of masses. The system was calibrated first without the weight dependent brake attached at all. Consequently, we applied the brake unit without additional mass and continued adding known masses. The behavior appeared non-linear between increasing RPM, but the slope in dependence of weight was still linear within the same RPM.
