# Supplementary figures and images for: Magnetic Resonance-Compatible Arm-Crank Ergometry: A New Platform Linking Whole-Body Calorimetry to Upper-Extremity Biomechanics and Arm Muscle Metabolism
Source: Front Physiol. 2021 Feb 19;12:599514. doi: 10.3389/fphys.2021.599514 (PMC7933461; doi:10.3389/fphys.2021.599514)

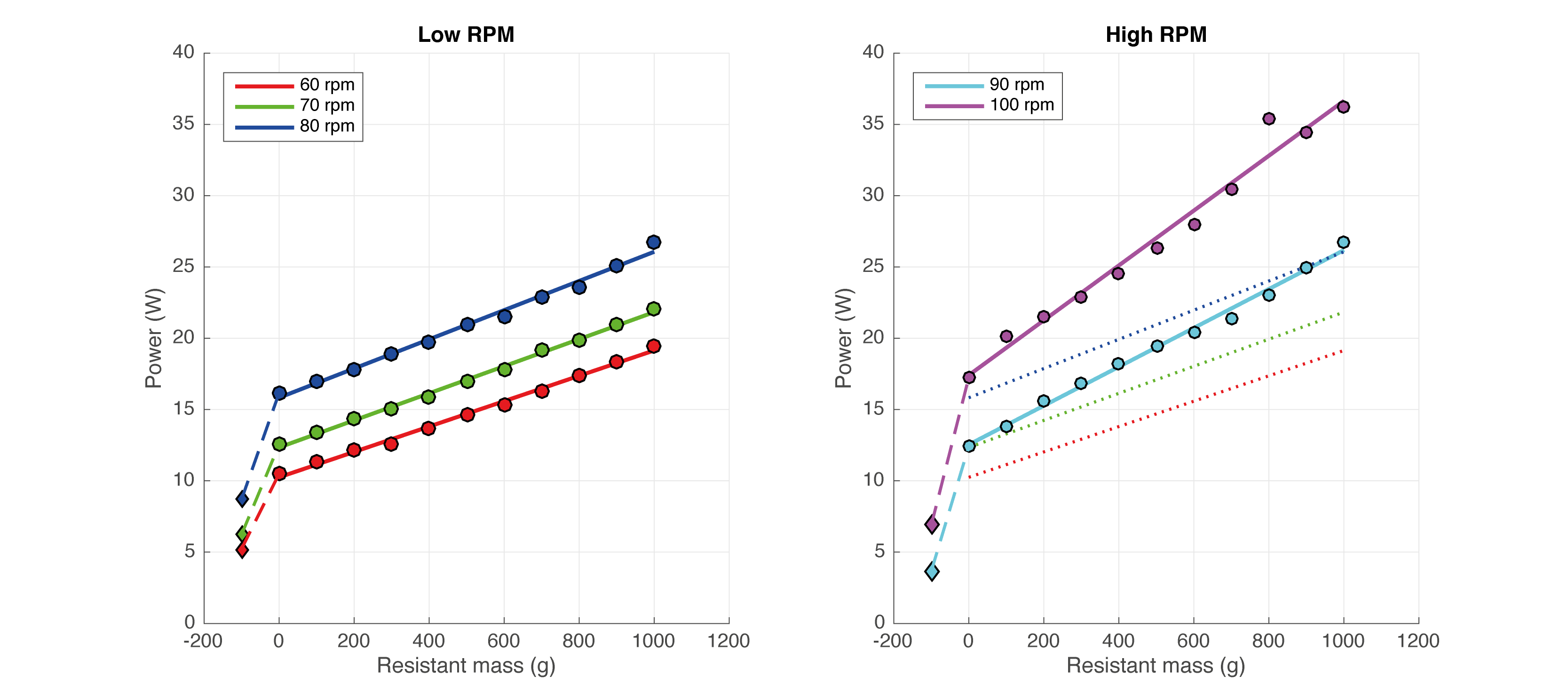

Supplement: Supplementary Figure 1 — The rolling resistance of the platform is manipulated by adding mass to a friction dependent brake on the flywheel. The consequent RPM-dependent power output (W) was measured by the in-factory lode calibrator and shows good linearity within a range of masses. The system was calibrated first without the weight dependent brake attached at all. Consequently, we applied the brake unit without additional mass and continued adding known masses. The behavior appeared non-linear between increasing RPM, but the slope in dependence of weight was still linear within the same RPM. [file Image_1.TIFF]
